# Improving phosphorus sustainability of sugarcane production in Brazil

**DOI:** 10.1111/gcbb.12650

**Published:** 2019-10-18

**Authors:** Amin Soltangheisi, Paul J. A. Withers, Paulo Sergio Pavinato, Maurício Roberto Cherubin, Raffaella Rossetto, Janaina Braga Do Carmo, Gustavo Casoni da Rocha, Luiz Antonio Martinelli

**Affiliations:** ^1^ Laboratory of Isotope Ecology Center for Nuclear Energy in Agriculture University of São Paulo Piracicaba Brazil; ^2^ Lancaster Environment Centre Lancaster University Lancaster UK; ^3^ Department of Soil Science Escola Superior de Agricultura Luiz de Queiroz University of São Paulo Piracicaba Brazil; ^4^ Agência Paulista de Tecnologia dos Agronegócio Piracicaba Brazil; ^5^ Department of Environmental Sciences Federal University of São Carlos Sorocaba Brazil; ^6^ Faculdade de Ciências Agronômicas Universidade Estadual Paulista Botucatu Brazil

**Keywords:** 5R P stewardship, bioethanol, Brazil, nutrient efficiency, phosphorus, sustainability

## Abstract

Phosphorus (P) use in global food and bioenergy production needs to become more efficient and sustainable to reduce environmental impacts and conserve a finite and critical resource (Carpenter & Bennett, *Environmental Research Letters*, 2011, *6*, 014009; Springmann et al., *Nature*, 2018, *562*, 519). Sugarcane is one crop with a large P footprint because production is centered on P‐fixing soils with low P availability (Roy et al., *Nature Plants*, 2016, *2*, 16043; Withers et al., *Scientific Reports*, 2018, *8*, 2537). As global demand for processed sugar and bioethanol continues to increase, we advocate that improving P efficiency could become a key sustainability goal for the sugarcane industry. Here, we applied the 5R global P stewardship framework (Withers et al., *Ambio*, 2015, *44*, 193) to identify more sustainable options to manage P in Brazilian sugarcane production. We show that current inputs of P fertilizer to the current crop area could be reduced by over 305 Gg, or 63%, over the next three decades by reducing unnecessary P fertilizer use, better utilization of recyclable bioresources and redesigning recommendation systems. Adoption of these 5R options would save the sugarcane industry in Brazil 528 US$ million and help safeguard global food and energy security.

## Graphical abstract

Improving phosphorus efficiency could become a key sustainability goal for the sugarcane industry. Here, we applied the 5R global phosphorus stewardship framework to identify more sustainable options to manage phosphorus in Brazilian sugarcane production. We show that current inputs of phosphorus fertilizer to this crop could be reduced by over 305 Gg, or 63%, over the next three decades by reducing unnecessary phosphorus fertilizer use, better utilization of recyclable bioresources and redesigning recommendation systems. Adoption of these 5R options would save the sugarcane industry in Brazil 528 US$ million and help safeguard global food and energy security.

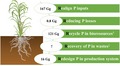

## 1 INTRODUCTION

**Figure 1:**
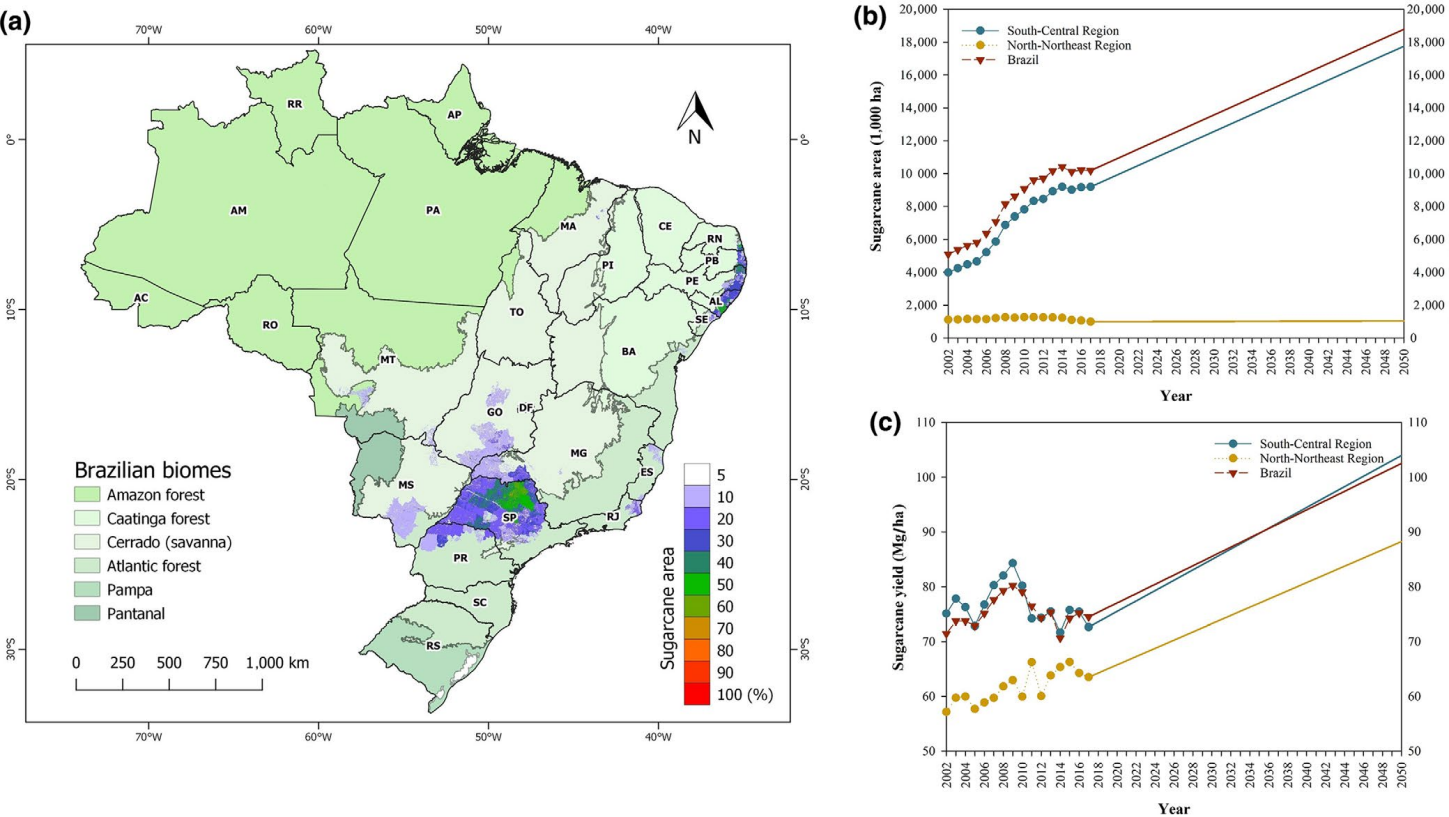
Sugarcane cultivated area in percentage of pixel area (resolution 1 × 1 km), highlighting the two largest sugarcane‐producing regions (South‐Central and North‐Northeast) (a). Sugarcane cultivation map was built from updated dataset provided by Dias, Pimenta, Santos, Costa, and Ladle (2016) (http://www.biosfera.dea.ufv.br). Current and future (2019–2050) trends in Brazil's sugarcane area (b) and yield (c)

Sugarcane (*Saccharum spp*) is the largest source of processed sugar and bioethanol, and Brazil is the largest producer with 10 Mha (CONAB, 2019) supplying 40% of total global sugarcane production (FAOSTAT, 2017), accounting for 20% of global sugar consumption and 90% of global sugarcane bioethanol production (OECD, 2015). The crop occupies 16% of the agricultural land in Brazil, mainly in the South‐Central region (90%) and along the coastline in the Northeast and North (10%; Figure 1). Rapid expansion of the crop area (>100% increase since 2002) and a doubling of average stalk yields (from 37 to 75 Mg/ha since 1975) due to improved soil quality, plant breeding, and crop agronomy (Otto et al., 2016) have dramatically increased total sugarcane stalk production from 90 Tg in 1975 to 620 Tg in 2019 (CONAB, 2019). Sugar exports and bioethanol use contribute about 10% to Brazil's agricultural economy, and further expansion of this important crop is forecast to meet the increasing global demand for sugar and bioethanol (OECD, 2015). Assuming current levels of crop expansion and yield improvements, the sugarcane area will be 18.8 Mha in 2050 producing a total stalk yield of 1,937 Tg (Figure 1).

As with many other crops in Brazil, a major constraint to sugarcane production is the low availability of phosphorus (P) in the highly P‐fixing soils. Three‐quarters of global croplands with high P‐fixing soils are located in Brazil, and large amounts of inorganic P fertilizer are needed to help overcome soil P fixation capacity and supply sufficient available P to optimize crop growth and development (Roy et al., 2016). This inefficiency in P use means that the crop has a large P footprint and receives 20% (350 Gg) of Brazil's total consumption of highly soluble inorganic P fertilizer (FAOSTAT, 2017). Since Brazil's own reserves of mineable phosphate rock (PR) are of relatively poor quality and currently limited to only c. 50 years supply (Withers et al., 2018), the country is heavily dependent on fertilizer imports (60% of national consumption according to ANDA, 2017). With such a large P footprint, the security of sugarcane production in Brazil is vulnerable to a future P scarcity, or a large increase in market prices, as occurred in 2008, when the price of PR increased over 800% (Mew, 2016). Phosphorus use on the crop could become more efficient and sustainable to help safeguard food and bioenergy security, avoid any adverse environmental impacts on water quality, reduce reliance on fossil fuels, and help preserve a finite and critical global resource (Cordell & White, 2014; Jarvie et al., 2015; Withers et al., 2018).

Here we examined the efficiency of P use in Brazilian sugarcane production and applied the 5R global P sustainability framework proposed by Withers et al. (2015) to help prioritize more sustainable options to manage P, and reduce the crops dependency on imported inorganic P fertilizer. The 5R framework considered the opportunity to **R**ealign P inputs to more precisely match the P demand of sugarcane (1R), **R**educe P losses to water (2R), **R**ecycle existing bioresources more effectively (3R), **R**ecover and reuse P from waste where feasible (4R), and **R**edesign production systems to improve the P sustainability of sugarcane production (5R). Using this framework, we quantified the potential savings in costly imported manufactured P that might be achievable over the next three decades, and what future research is required to help facilitate the transition toward more P sustainable production systems.

## 2 PHOSPHORUS DEMAND AND EFFICIENCY OF USE

**Figure 2:**
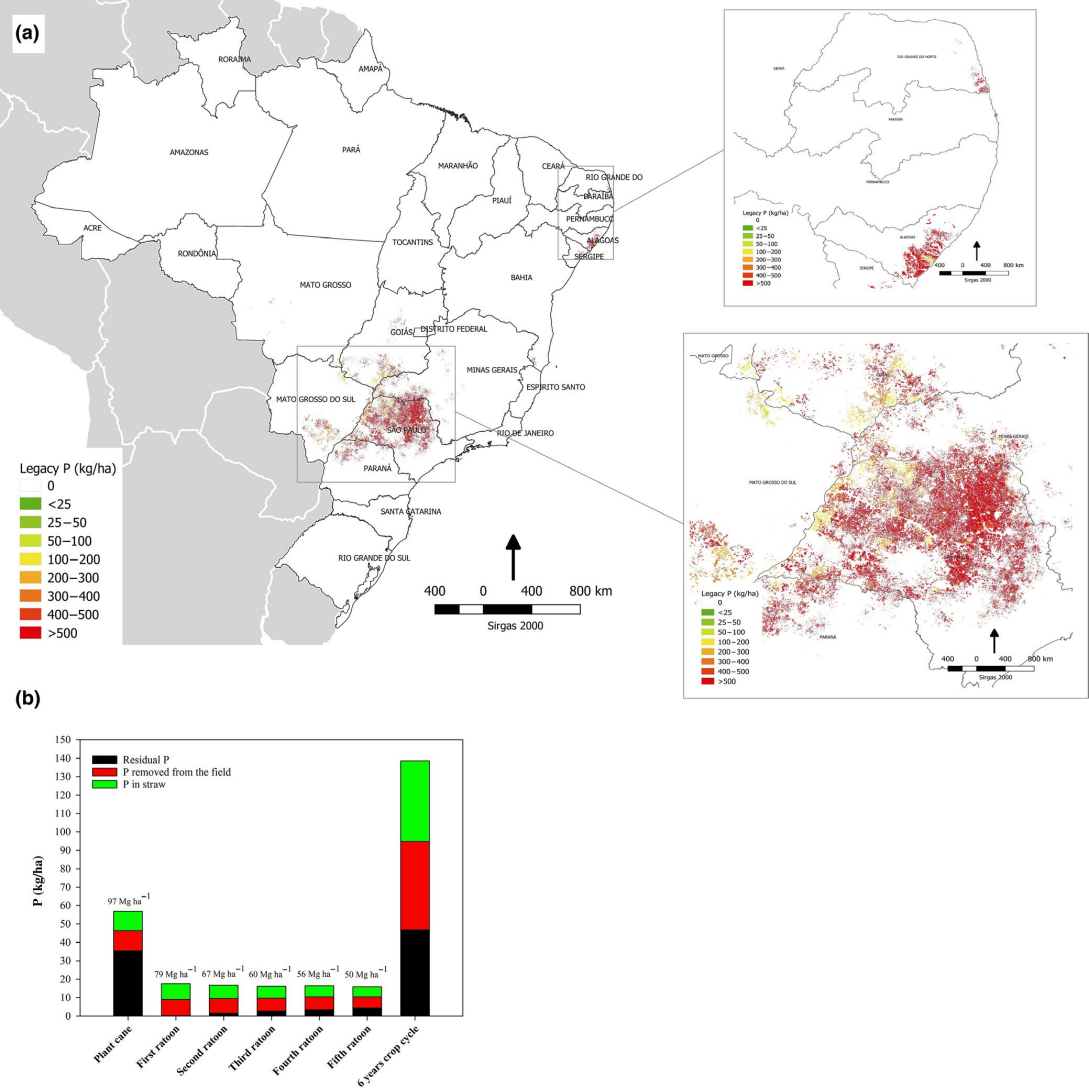
Map of legacy P in sugarcane fields of North‐Northeast and South‐Central regions of Brazil (a); Residual P, P removed from the field, and P in straw in six sugarcane life cycles averaged among Brazilian sugarcane fields (b). Number at the top of each bar shows the average stalk yield of that crop cycle

Sugarcane is a C_4_ plant harvested annually but with a long production cycle of 5–7 years. Nationally recommended P inputs at crop establishment range from 26 to 52 kg P/ha depending on soil test P (STP) level (usually measured by anion exchange resin), with a further 13 kg P/ha recommended in subsequent years (usually from second ratoon onward) under normal conditions (Raij, Cantarella, Quaggio, & Furlani, 1997). Over a 6 year growing cycle, these recommended fertilizer P inputs average almost 27 kg P ha^−1^ year^−1^. Actual inorganic P fertilizer use on sugarcane in Brazil is typically 50–80 kg P/ha at crop establishment and averages 35 kg P ha^−1^ year^−1^ overall (CONAB, 2019). However, average P export in sugar stalks removed from the field over the 6 year growing cycle is only 11 kg P/ha (Figure 2). This crop recovery of added P fertilizer (41%) leaves large residues in the soil to build up background P fertility. However, if allowed to continue beyond levels which are considered agronomically useful, this soil P accumulation is not only an unnecessary waste of a critical resource but will eventually pose a long‐term risk to water quality (Carpenter & Bennett, 2011; Springmann et al., 2018; Withers et al., 2019). We estimated that the legacy of residual P that has accumulated in the soil since the crop was first cultivated is approximately 4 Tg (Figure 2), and this legacy P could be better utilized to improve the resilience of the sugarcane crop to future P shocks (Rodrigues, Pavinato, Withers, Teles, & Herrera, 2016; Rowe et al., 2016). This legacy P is largely located in the São Paulo region where sugarcane expansion and fertilizer P inputs have been the greatest. With a theoretical yield potential of 200 Mg/ha (Dias & Sentelhas, 2018), and as the crop area in Brazil continues to expand by 0.12 Mha/year (Filoso et al., 2015), demand for inorganic P fertilizer will reach 480 Gg by 2050 at current rates of P fertilizer use, if more sustainable management options to improve crop P efficiency are not implemented. This high dependency on P fertilizer threatens the country's future food and bioenergy security.

## 3 5R OPTIONS FOR THE SUSTAINABLE MANAGEMENT OF P IN SUGARCANE

### 3.1 Realign P inputs (1R)

In many areas of production, overuse of fertilizer P to correct perceived poor soil P availability continues regardless of soil P fertility level. In other areas, P fertilizer is only applied when cane yields start to decline (Landell et al., 2003; Zambrosi, 2012). Considerable yield gaps, therefore, exist across Brazil: for example, in Northeast Brazil, yields remain much lower (c. 60 Mg/ha) than the national average due to the lack of regular rainfall, lower levels of mechanization, poor soils, and poorer management (CONAB, 2019). There is, therefore, large potential to realign P inputs to more closely match crop P demand, overcome yield gaps, and improve crop recovery of added P through implementation of P fertilizer stewardship (right rate, timing, method, and source; IFA, 2009) and correcting other limitations to yield (e.g., soil acidity, irrigation, and supply of other nutrients; Bordonal et al., 2018).

Best practice for timing and method of fertilizer P application to sugarcane is now well established. Fertilizers and any manures are typically placed as a single dose in the furrow at 8–10 cm depth and close to the planted seedling, with broadcast P topdressings to ratoons in subsequent years. Broadcast or incorporated P facilitates more contact with the soil and is most effective at raising overall soil P fertility (Rossetto, Farhat, Furlan, Gil, & Silva, 2002; Vitti & Mazza, 2002), and split applications with some in the furrow and some broadcast has given higher yields in some experiments (Albuquerque, Sá, Rodrigues, Moura, & Oliveira Filho, 2016). However, the furrow application remains the most practical and cheapest. Foliar application of soluble P sources has been suggested as a complementary management strategy to enhance early growth on P‐deficient soils (e.g., Zambrosi & Mesquita, 2016) but is not designed to substitute for P application to the soil. The largest opportunity for realigning P inputs to maximize efficiency, therefore, rests with rates and sources of P.

Once agronomically optimum threshold STP levels have been reached (15 mg/kg resin P), annual P inputs no longer need to exceed crop P demand by more than 10% (Raij et al., 1997). For example, recent research on tropical soils has shown that P fixation processes are greatly reduced and efficiency of P use increases once soils have become sufficiently saturated to block P adsorption pathways (Barrow & Debnath, 2014). Taking into account the distribution of STP concentrations in sugarcane fields, the rate at which STP builds up in soils, and the fertilizer P replacement rates required to match crop P offtake (+10%), we estimate that c. 30% of the sugarcane area can make immediate savings amounting to 50 Gg of P (see Methods). A further 50 and 67 Gg of fertilizer P can be saved in 9 and 15 years, respectively, when resin P levels have attained required threshold levels.

Although sugarcane growers have traditionally relied on highly water‐soluble P fertilizers to maximize P availability to the crop, alternative less expensive and more slow‐release inorganic and organic sources of P can substitute for imported P (see Table S1). For example, research has shown that crop yield and sugar quality are not compromised by mixed 50:50 applications of rock phosphate and triple superphosphate (TSP) compared to TSP alone (Cantarella, Rossetto, Landell, Bidoia, & Vasconcelos, 2002; Rossetto et al., 2002). We estimate that 46 Gg of imported soluble P fertilizer could be saved by substituting much cheaper RP for inorganic soluble P imports at crop establishment on both P‐deficient and P‐sufficient soils (see Methods). While not achieving a saving in total P inputs, the costs of production are reduced by an estimated 10 US$ million. The future development of new technology fertilizers may also help to overcome P fixation and improve efficiency (Bordonal et al., 2018), but research is not yet sufficiently established to allow quantification of potential P savings.

### 3.2 Reducing phosphorus losses (2R)

Soil degradation caused by erosion and compaction is a major problem in sugarcane fields in Brazil, especially when the crop is burnt before harvest (Hartemink, 2008; Politano & Pissarra, 2005). For example, Sparovek and Schung (2001) estimated soil losses in sugarcane production in São Paulo state of up to 30 Mg ha^−1^ year^−1^, while they did not exceed 2 Mg ha^−1^ year^−1^ from forests and pastures. In addition to the loss of crop production potential, soil losses lead to P losses and reduced overall P use efficiency (Izidorio, Martins Filho, Marques Júnior, Souza, & Pereira, 2005; Paula, Martins Filho, Farias, & Siqueira, 2016; Politano & Pissarra, 2005). Critical periods for increased erosion risk are during the initial conversion of pasture to sugarcane when the grass is desiccated, the period between crop harvesting and regrowth, and at replanting when soils can remain bare for several months (Martinelli & Filoso, 2008). Paula et al. (2016) considered a minimum soil surface coverage of 42% was crucial to reduce the clay content, and consequently the P content, of eroded sediments. We estimate national P losses associated with erosion in sugarcane production under current practices at 4 Gg in 2018 rising to 7.2 Gg by 2050 (see Methods).

As preharvest burning is now being phased out in mechanizable areas (defined as lands with slopes lower than 12%), and has been reduced by over 60% in São Paulo state since 2006 (Aguiar, Rudorff, Silva, Adami, & Mello, 2011), straw residues can be left in the field (referred to as the green harvest system) to reduce erosion risk, conserve soil moisture, and build up soil organic matter. Considering the average yield of 80 Mg/ha, approximately 14.1 Mg/ha of the straw dry matter remains on the soil surface after each harvest (Bordonal et al., 2018; Figueiredo & La Scala Jr, 2011). For example, Andrade, Martins Filho, Torres, Pereira, and Marques Júnior (2011) observed that P losses in a green‐cane trash blanketing (GCTB) system were 60% less than when sugarcane was burnt, and Martins Filho, Liccioti, Pereira, Marques Júnior, and Sanchez (2009) found that retaining 50% and 100% of straw on the soil surface reduced erosion by 70% and 90%, respectively, in comparison with a bare soil.

However, sugarcane straw is also a source of biofuel to provide electricity for the sugarcane mills and provides an economic return to the grower when removed from the field. Retention of too much straw on the soil surface also has some disadvantages: it hinders effective mechanical cultivation (Magalhães et al., 2012) and fertilizer incorporation (Bianchini et al., 2014), increases the risk of fire during very dry periods (Rossetto, Cantarella, Dias, Landell, & Vitti, 2008), reduces initial crop tillering (Lisboa et al., 2018), and encourages pest and disease infestations (Castro et al., 2019). A sustainable option is to retain 6–8 Mg/ha of the straw residue in the field to provide both economic and environmental benefit and lessen any agronomic disadvantage (Carvalho et al., 2017). We estimate that increased adoption of GCTB in South‐Central and North‐Northeast regions would enable P loss savings of 0.8 Gg of P by 2050 (see Methods).

### 3.3 Recycling (3R) and recovery (4R) of P bioresources

The processing of each metric ton of sugarcane to produce sugar and ethanol in sugar mills produces ~35 kg of filter cake (FC, 30% dry matter), a by‐product which can be beneficially reused in sugarcane fields in its natural state or by composting (Prado, Caione, & Campos, 2013). We estimate that 65 Gg of P as FC is currently being produced in Brazil, and this is predicted to increase to 110 Gg of P, or ~23% of sugarcane P demand by the year 2050 (see Methods). Phosphorus in FC is mostly organic and must be mineralized over two or three seasons to supply P for plant uptake, but research suggests that FC can be partially or fully substituted for inorganic P fertilizer at planting without confounding crop yields or the build‐up rates of soil P fertility (Caione et al., 2015; Elsayed, Babiker, Abdelmalik, Mukhtar, & Montange, 2008). Additional benefits in conserving soil moisture and microbial diversity have also been observed; for example, Arruda et al. (2019) found that FC modified the structure of fungal and bacterial communities, whereas only bacterial and archaea communities were influenced by mineral P fertilizer use. Realizing effective substitution is dependent on the cost of transporting FC to surrounding farmland, and recycling distances from the mills are currently 20–30 km. Assuming a conservative 50% substitution value taking into account immediate P availability (Raij et al., 1997), the saving in P fertilizer inputs is currently estimated as 55 Gg by using FC (see Methods).

Another by‐product of the sugarcane biofuel industry that is currently recycled back to the field as an organic amendment by fertigation is a liquid effluent called vinasse (Filoso et al., 2015; Gunkel et al., 2007). However, the P content is relatively low which limits its substitution value (50 Mg P by 2050, see Methods) and a high potassium content further limits application rates (Technical Standard P4231, 2005). Decomposition of straw residues left in the field also provides P for crop uptake. We estimate that maintaining 50% of the straw in the field to combat erosion risk would release 66 Gg of P in 2050, covering 9.5% and 38% of sugarcane P demand for the next plant cane and ratoon, respectively (see Methods).

Municipal wastewater biosolids and manures from concentrated animal feeding operations (CAFOs) are also potential secondary biosources for recycling P in Brazil (Powers et al., 2019; Withers et al., 2018). Trimmer and Guest (2018) estimated that c. 7 Gg of P was generated annually by the population of São Paulo city alone, but in reality, only about 15% of wastewater P collected and treated in Brazil is reapplied to agricultural land as biosolids (Andreoli, Garbossa, Lupatini, & Pegorini, 2008). Wastewater biosolid P is also of limited bioavailability (<25%) to plants compared to other bioresources because of precipitation with iron during sewage treatment (Krogstad, Sogn, Asdal, & Saebo, 2005; O'Connor, Sarkar, Brinton, Elliot, & Martin, 2004). Similarly, most CAFOs are concentrated in areas of Brazil that are too far from the main sugarcane areas to make it economically feasible to transport livestock manures for recycling to land. There are also concerns over disease transfer which add to the treatment costs of making these bioresources safe to apply; for example, salmonella transfer in poultry manure (Penakalapati et al., 2017).

However, there is large potential to recover P from wastewaters and CAFO manures in inorganic forms that are more transportable and bioavailable for reuse in agriculture; for example, Brazil has 7.2% of the world's recoverable total (i.e., humans plus animals) fecal biomass (Berendes, Yang, Lai, Hu, & Brown, 2018). However, this requires investment in new technologies, regulatory compliance, the development of markets to trade them, and research to demonstrate their on‐farm fertilizer substitution value (Withers et al., 2015).

### 3.4 Redesign sugarcane production systems (5R)

#### 3.4.1 Prediction of fertilizer requirement

**Figure 3:**
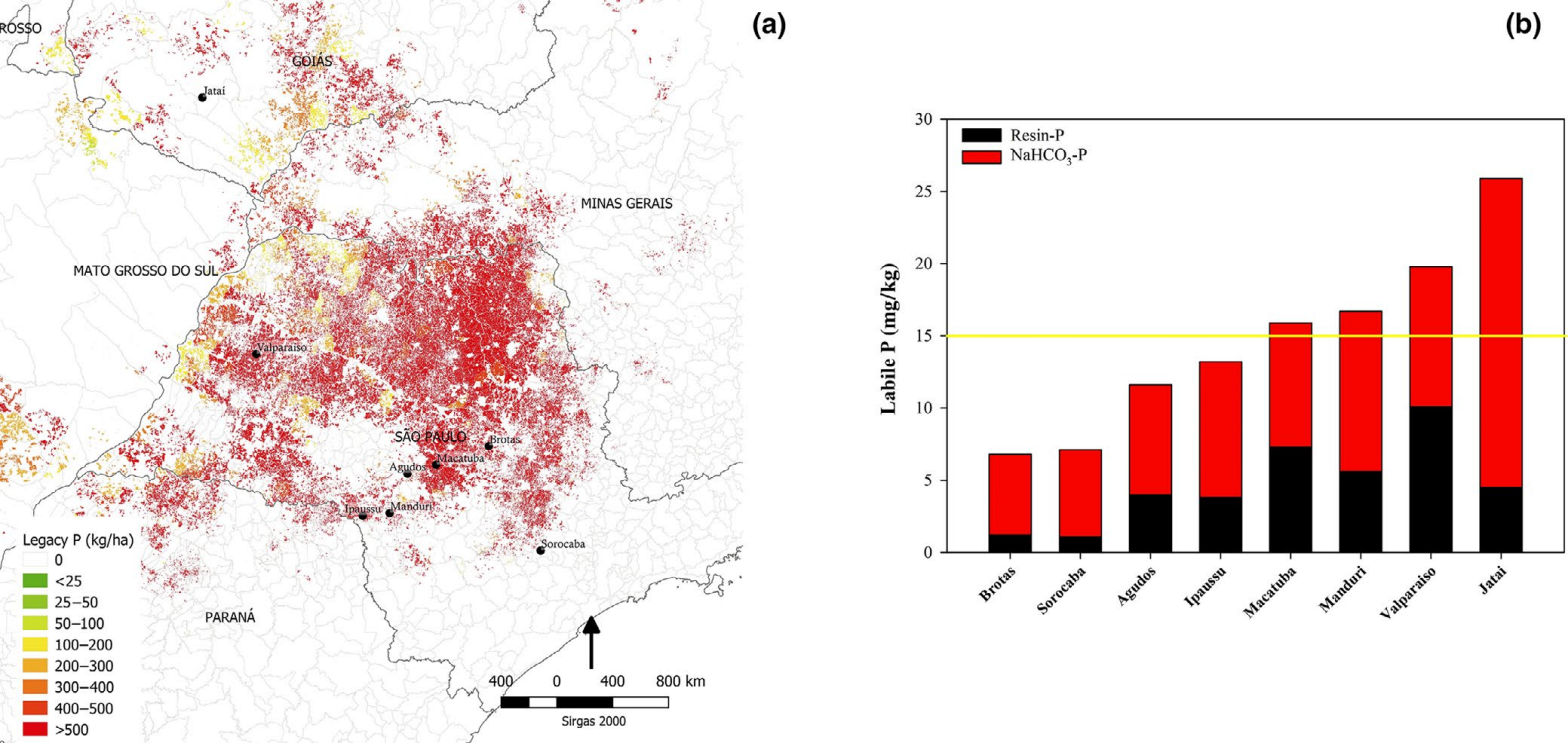
Map of eight sites investigated in the states of São Paulo and Goiás (a), and resin P and P extracted by 0.5 M NaHCO_3_ in each site (b). Sum of resin P and P extracted by 0.5 M NaHCO_3_ is considered as inorganic labile P

Currently, fertilizer P requirements for sugarcane in Brazil are dependent on STP analysis by resin, with a recommended threshold level of 15 mg/kg needed to optimize crop yield (Raij et al., 1997). However, regional experience suggests that sugarcane is yielding well above average (>100 t/ha) on soils with <15 mg/kg resin‐P, for example, in the states of São Paulo and Goiás. This is supported by recent evidence from a replicated P response trial on a clayey oxisol (Macatuba) in São Paulo state where plant cane and first ratoon crops yielding >150 and >100 Mg/ha, respectively, did not show any yield response to P fertilizer (180 kg P/ha as TSP) even though resin P was only 7 mg/kg (Soltangheisi et al., 2019; Figure 3).

As resin extracts only a small proportion of the total reserves of unused P that accumulate in Brazilian soils, this extractant maybe underestimating the amounts of soil labile P that sugarcane can exploit. Soltangheisi et al. (2019) observed that in sugarcane fields regularly fertilized with TSP, only 1.4% and 5.6% of total P were extracted by resin in clayey and sandy soils, respectively, while 6.0% and 18.0% were extracted by resin plus 0.5 M NaHCO_3_ (inorganic labile P). Since the amounts of P extracted by resin plus 0.5 M NaHCO_3_ are both considered plant available (Hedley, Stewart, & Chauhan, 1982; Tiessen & Moir, 1993), a fertilizer requirement prediction based on resin alone may overestimate the fertilizer P requirement. At the Macatuba field trial, labile P (16 mg/kg) was more than twice resin P (Soltangheisi et al., 2019), what was the case also for the other seven sites evaluated here (Figure 3b). At another sandy soil site in São Paulo state (Agudos), where the concentration of both resin P and inorganic labile P was <15 mg/kg, there was a significant (*p* < .05) response in plant cane and first ratoon yield of 10.8 and 18.8 Mg/ha to P fertilizer (180 kg P/ha as TSP; Soltangheisi et al., 2019). We estimate a 16 Gg P immediate saving in P fertilizer inputs if national recommendation systems adopt inorganic labile P instead of resin P (see Methods).

#### 3.4.2 Agroengineering

Sugarcane varieties show some variation in yield response to applied P and P rates could be slightly adjusted by carefully matching choice of cultivar to environmental conditions; for example, Silva Calheiros et al. (2012) found that 84 and 77 kg/ha P were required at establishment for RB867515 and RB92579 varieties on a P‐deficient soil, respectively. Cultivars which are more efficient in soil P acquisition, and can translocate P more efficiently within the plant, on P‐deficient soils have also been identified (Arruda et al., 2016; Zambrosi, Ribeiro, Machado, & Garcia, 2017), but they still require fertilizer P inputs to optimize yield. Further research is required to assess the feasibility of introducing P‐efficiency genes into high‐yielding cultivars of sugarcane to lower their P fertilizer requirements. Similarly, P‐solubilizing bacteria (e.g., *Agrobacterium radiobacter*, *Bacillus* *megaterium*; Shankaraiah, Hunsigi, & Nagaraju, 2000), *Acidithiobacillus* oxidizing bacteria (Stamford, Lima, Lira, & Santos, 2008), and phosphobacteria (Ramesh, Chinnusamy, & Jayanthi, 2002), and mycorrhizal associations (e.g., *Aspergillus awamori*; Shankaraiah et al., 2000) have been shown to enhance P availability to sugarcane in P‐deficient soils, but their ability to offset crop P fertilizer inputs still needs to be proven (Gopalasundaram, Bhaskaran, & Rakkiyappan, 2012). For example, Schütz et al. (2018) showed that microbial P solubilizers and arbuscular mycorrhizal fungi can enhance yield by 15% in tropical soils with low levels of plant available P. Research is needed to identify microbial strains that can tolerate P fertilizer inputs while facilitating soil P mobilization and plant uptake, and to develop more integrated practices that combine crop and microbial engineering with lower and more targeted P fertilizer inputs (Rowe et al., 2016; Withers et al., 2018).

## 4 CHALLENGES TO IMPLEMENT 5R P STEWARDSHIP FRAMEWORK IN BRAZILIAN SUGARCANE PRODUCTION

Our findings suggest that the 5R P stewardship framework is a key strategy for improving the efficiency and sustainability of P management in the Brazilian sugarcane industry. However, implementation of 5R options requires an acceptance by sugarcane growers and processers of the need for P sustainability, and changing practices to meet international sustainability goals for future food and bioenergy security against a backdrop of an industry recession, particularly in North‐Northeast regions, will be challenging. Raising awareness of P vulnerability and the economic and environmental benefits of sustainable P use within the industry will be an important first step toward the necessary transitions in industry practices (Jacobs, Cordell, Chin, & Rowe, 2017). Win‐win practices with synergistic benefits are likely to be most palatable, for example, the dual benefits of lowering fertilizer P inputs and reducing soil erosion by recycling straw and filter cake residues. However, phasing‐out sugarcane burning and transition from manual to mechanized harvesting systems will depend on the fiscal conditions of the growers, and revenue generation from selling electricity produced from sugarcane straw may outweigh any agronomic advantage, especially during the dry season when hydroelectric power output is low. Improved, science‐based regional guidelines for recycling all bioresources produced in sugarcane production (straw, filter cake, and vinasse) will help to build confidence in the use of these materials as fertilizer substitutes, and encourage growers to move away from traditional practices that place an overreliance on P fertilizer. For example, filter cake and vinasse could be enriched with micro‐ and other macronutrients to make them more economically viable to transfer to fields further than 30 km from the mill. Isolating plant genes that can improve P utilization efficiency and the development of P‐efficient sugarcane varieties that require less P input and can perform well on P‐fixing soils remains a key scientific challenge. However, the impetus for such advances in crop science has not yet become accepted. The new STP methods proposed here do not pose any analytical challenges, but require full‐field calibration before their introduction into soil testing laboratories. Such field calibration is time consuming and expensive, but will provide longer term benefits for farm profitability in reducing the application of unnecessary P fertilizer. The economic and sustainability case for making such transitions needs to be crystallized and accepted by the sugarcane industry. Government incentives toward such sustainable transitions in support of international sustainability goals may be required.

## 5 CONCLUSION

**Figure 4:**
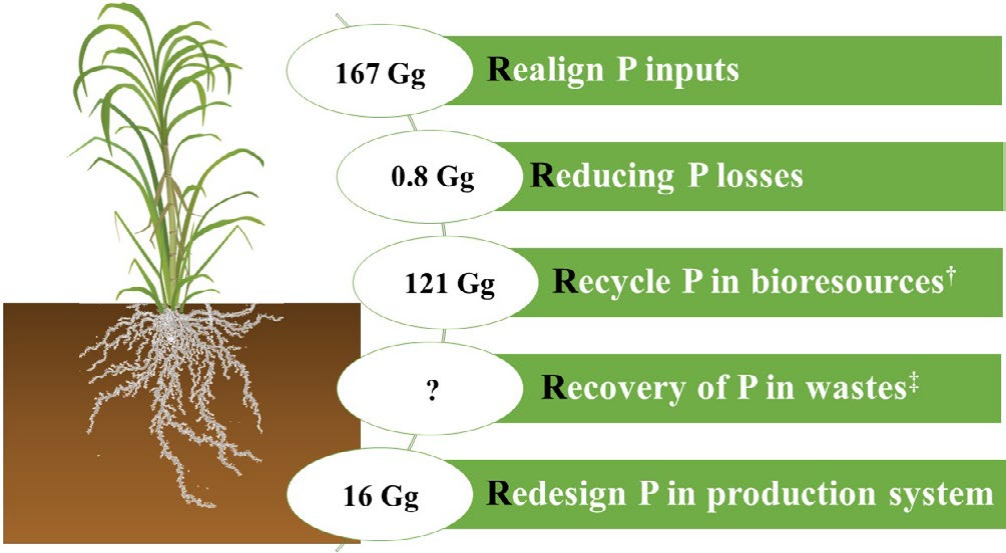
Potential of 5R strategy to increase P sustainability of sugarcane production in Brazil by 2050. ^†^Bioresources including filter cake (55 Gg of P), straw (66 Gg of P), and vinasse (50 Mg of P). ^‡^There is a large potential for P recovery from wastes (e.g., municipality wastewater biosolids and manures) in Brazil, but it is negligible in the current situation

Global food production must become more sustainable to alleviate food poverty, reduce environmental damage, and conserve vital resources for future generations. Per capita consumption of processed sugar is increasing globally and sugarcane will remain the dominant source (currently 86%). In Brazil, the crop is also an important source of bioethanol and bioelectricity which has driven the recent rapid expansion of the crop. Managing P more efficiently could become a key sustainability goal for the sugarcane industry as we have identified considerable scope to reduce P fertilizer inputs from their current excessive levels without compromising crop yield or quality by considering the 5R global sustainability framework (Figure 4). Largest opportunities to reduce the crops P footprint lie in matching fertilizer inputs to current cropland area more closely to actual P demand, utilizing legacy soil P reserves where these have exceeded their agronomic optimum, recycling of the industry's filter cake and straw residues more effectively, and redesigning recommendation systems to improve prediction of fertilizer needs. The potential savings to the sugarcane industry in Brazil are 528 US$ million and would increase the profitability of the crop as a replacement for fossil fuels. Application of this 5R framework should be extended to other global food commodities to increase the feasibility of sustainable global intensification.

## 6 METHODS

***Fertilizer usage:*** To calculate the monetary value of P saving using 5R, a P fertilizer price of $1.7 kg^−1^ P was used.

### 6.1 Realign P inputs (1R)

In line with many national fertilizer recommendations systems, we assume that the sugarcane crop requires only a Maintenance (M) + 10% P application to satisfy the demand for growth once critical STP levels have been reached (15 mg/kg of resin P for Brazilian sugarcane; Barrow & Debnath, 2014). According to available data on resin P concentrations in Brazilian sugarcane fields in São Paulo region, c. 30% of the area has resin P concentrations greater than 15 mg/kg. As M + 10% is only 11 kg/ha relative to the current annual P application rate of 27 kg/ha averaged over the 6 year crop life cycle, 16 kg P ha^−1^ year^−1^ of fertilizer P can be saved immediately amounting to a total of 50 Gg of P at the country scale. Currently, 40% and 30% of the sugarcane area have resin P concentrations less than 5 and 10 mg/kg, respectively, and these areas will reach the critical threshold resin P level in 15 and 9 years, respectively, according to the eight different sites with different total and resin P contents investigated here (Figure 3; Table S2). When these areas reach the critical resin P threshold, a further 67 and 50 Gg of P can be saved. The calculations are based on a 20 cm soil depth consistent with the depth of cultivation and the national fertilizer recommendation system.

To calculate the monetary value of using RP instead of TSP in Brazilian sugarcane, price of RP and TSP were considered as 216 and 430 US$ per Mg, respectively, in September 2019. This RP is from sedimentary rocks mostly imported from Morocco, Tunisia, Israel, and Peru.

### 6.2 Reducing phosphorus losses (2R)

***Erosion:*** National P loss associated with erosion in sugarcane production under current practices was based on 1.14 Mha of sugarcane in Brazil still being harvested by burning (CONAB, 2019) with an average P loss rate of 1.07 kg P/ha (Izidorio et al., 2005), and the remainder (9.04 Mha) green harvested with 50% of the straw residue left on the soil surface and a P loss rate of 0.32 kg P/ha (Martins Filho et al., 2009). Planned crop expansion would increase P loss by erosion to 7.2 Gg by 2050 under current practice, but increased adoption of GCTB in South‐Central and North‐Northeast regions by 100% and 48%, respectively, would reduce this national loss to 6.4 Gg, a saving of 0.8 Gg of P to be retained in the soil to maintain soil resources and P fertility.

### 6.3 Recycle Bioresources (3R)

***Filter cake*** *:* Considering the P content of FC of 8 g/kg and total FC production of 8.07 Tg dry solids (Prado et al., 2013), approximately 64.9 Gg of P is currently being produced in Brazil. This is predicted to increase to 110 Gg of P, or ~23% of sugarcane P demand by the year 2050. We assume 50% P availability immediately after FC application.

***Vinasse*** *:* Considering the production of 64 billion liters of ethanol in Brazil by the year 2050 (OECD, 2015), 837 billion liters of vinasse will be generated at that time containing 50 Mg P. Each cubic meter of vinasse contains on average 60 mg P (Christofoletti, Escher, Correia, Marinho, & Fontanetti, 2013; Rossetto, Dias, & Vitti, 2008). The total potential saving in P by recycling vinasse is therefore 50 Mg P.

***Straw*** *:* Each kilogram of sugarcane straw on average contains 1.05 g P (Cherubin et al., 2018; Fortes, Trivelin, & Vitti, 2012). We consider straw as top leaves + bottom leaves. Top leaves (younger) add 5.7 kg P/ha which 70% (4 kg P/ha) of it is immediately plant available (Cherubin et al., 2019). Bottom leaves (older) add 1.78 kg P/ha which 50% (0.9 kg P/ha) of it is immediately plant available (Cherubin et al., 2019). Overall, we can say that from 7.5 kg P/ha added by straw to the soil, 4.9 kg P/ha is immediately plant available. With a cropland area of 18.8 Mha in 2050, the total potential saving in P by retaining 50% of the straw at harvest is therefore 66 Gg.

### 6.4 Redesign (5R)

Soil P fractionation analysis across sugarcane fields in São Paulo state suggested that crop available labile P is on average 20% greater than resin P in Brazilian oxisols (Figure S1). The potential saving in fertilizer P across the full 6 year growing cycle by adopting labile P as the indicator of P sufficiency across field trials assuming a similar threshold level of 15 mg/kg was 65.5 kg/ha (10.9 kg P ha^−1^ year^−1^). Extending this analysis by assuming that 20% of the sugarcane area in Brazil that was classed as deficient in P according to resin analysis (estimated at 70% of total area) is no longer deficient, then the difference in the recommended P input between deficient and not deficient situations over this area amounts to a 16 Gg P saving in P fertilizer inputs.

## AUTHOR CONTRIBUTIONS

A.S. and P.W. conceived the analysis and finalized all text; A.S. finalized all figures; A.S., P.P., M.C., and L.M. gathered the data; A.S., P.P., M.C., and G.R contributed to design the maps; all co‐authors contributed substantially to the main text; L.M. was the P.I. of the FAPESP project.
