# Supplementary material for: Improving phosphorus sustainability of sugarcane production in Brazil
Source: Glob Change Biol Bioenergy. 2019 Oct 18;11(12):1444–55. doi: 10.1111/gcbb.12650 (PMC6919938; doi:10.1111/gcbb.12650)
Supplement: Supplementary file 1 [file GCBB-11-1444-s001.pdf]

Supplementary Information for

Improving Phosphorus Sustainability of Sugarcane Production in Brazil

Amin Soltangheisi*[,a], Paul J. A. Withers[b], Paulo Sergio Pavinato[c], Mauricio Cherubin[c], Raffaella Rossetto[d], Janaina Braga Do Carmo[e], Gustavo Casoni da Rocha[f], Luiz Antonio Martinelli[a]

Author Affiliations:
[a]Departamento de Ecologia Isotópica, Centro de Energia Nuclear na Agricultura (CENA), Universidade de São Paulo (USP), Av. Centenário, 303 - São Dimas, CEP 13416-000, Piracicaba, SP, Brazil

[b]Lancaster Environment Centre, Lancaster University, Lancaster, UK

[c]Departamento de Ciência do Solo, Escola Superior de Agricultura Luiz de Queiroz, Universidade de São Paulo – ESALQ/USP, Av. Pádua Dias, 11, CEP 13418-900, Piracicaba-SP, Brazil

[d]Center-South Regional Pole, Paulista Agency for Agribusiness Technology (APTA), Rod. SP 127 km 30, 13400-970 Piracicaba, SP, Brazil

[e]Department of Environmental Sciences, Federal University of São Carlos (UFSCar), Rod. João Leme dos Santos Km 110, 18052-780 Sorocaba, SP, Brazil

[f]Faculdade de Ciências Agronômicas, Universidade Estadual Paulista (Unesp), Av. Universitária, nº 3780, CEP 18610-034, Altos do Paraíso, Botucatu, SP, Brazil

*To whom correspondence should be addressed: soltangheisi@usp.br

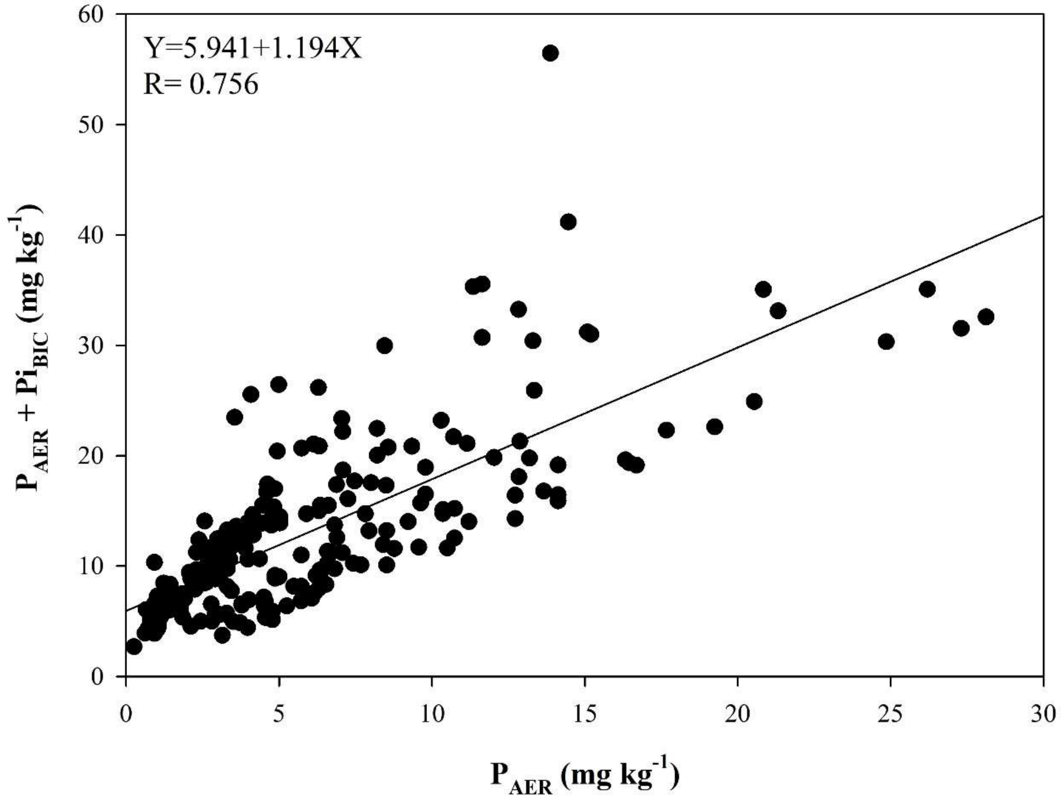

Supplementary Figure 1. Relationship between inorganic labile P (P_{AER} + Pi_{BIC}) and resin P (P_{AER}) in 8 different Brazilian sugarcane fields investigated in this study.

Supplementary Table 1. Advantages and disadvantages of different sources of phosphorus applied in Brazilian sugarcane fields

Phosphate Sources			Advantages	Disadvantages
Primary sources				
	Water soluble	MAP, DAP, SSP, TSP	• High initial agronomic efficiency<br>• High amount of water soluble P_2O_5	• Expensive<br>• More susceptible to loss via erosion
	Less soluble			
		Rock phosphate	• Less expensive<br>• Low P loss via leaching and erosion	• Releases P slowly suitable for areas with medium to high soil P levels<br>• Low agronomic efficiency in short term<br>• No water soluble P_2O_5
		Thermophosphate	• Contains magnesium, micronutrients and silicon<br>• Low P loss via leaching and erosion<br>• Low P fixation by Fe and Al (hydr)oxides due to the presence of silicon	• Expensive
	Biofertilizers		• Reduce P rates by 25-30%<br>• Replacing expensive sources of P with rock phosphate	• Difficulties associated with isolation and multiplication of pure strains of PSBs
Secondary sources				
	Filter cake		• Reduce the need for mineral fertilizers<br>• Contains 1.2-1.8% of P<br>• Contains 70% humidity which guarantees the sprouting of cane planted in winter	• It is free but the transport cost from the mill to the field is expensive<br>• Slow release P as it is mainly organic
	Vinasse		• Reduce the need for mineral fertilizers<br>• Provide water retention<br>• Increase organic matter and soil microflora	• It is free but the transport cost from the mill to the field is expensive<br>• Slow release P as it is organic<br>• Contains very high amounts of potassium<br>• Risk of salinization in rates higher than 400 m^3 ha^{-1}<br>• Causes delay of the maturation process in high quantities<br>• Contamination of ground water via leaching
	Straw		• Reduce the need for mineral fertilizers<br>• Reduces erosion<br>• Improve soil carbon content<br>• Provide water retention	

Supplementary Table 2. Soil phosphorus fractions in the sites investigated in this study

	Brotas†	Sorocaba†	Agudos‡	Ipaussu§	Macatuba‡	Manduri†	Valparaiso§	Jatai§
P_{AER}	1.2	1.1	4.0	3.8	7.3	5.6	10.1	4.5
Pi_{BIC}	5.6	6.0	7.6	9.4	8.6	11.1	9.7	21.4
Po_{BIC}	47.6	56.5	16.0	23.8	16.3	28.5	38.9	9.4
$Pi_{HID-0.1}$	23.1	22.9	21.0	83.4	18.5	32.1	28.1	62.3
$Po_{HID-0.1}$	45.7	109.2	45.5	42.7	94.1	183.0	89.1	72.6
P_{HCl}	1.3	0.8	32.5	14.8	11.5	2.6	14.2	4.5
$Pi_{HID-0.5}$	6.9	9.2	22.0	128.0	34.2	15.0	11.2	74.4
$Po_{HID-0.5}$	46.4	47.0	26.5	33.2	65.5	82.5	79.4	73.2
$P_{residual}$	77.0	418.0	76.9	667.2	274.5	1127.0	202.7	328.8
P_{total}	255.0	671.0	252.0	1006.5	530.5	1487.0	489.5	650.9

†unpublished data obtained by A. Soltangheisi

‡data from Soltangheisi et al. (2019)

§data from Cherubin et al. (2016)

P_{AER}: P extracted with anion exchange resin; Pi_{BIC} and Po_{BIC}: inorganic and organic P extracted with 0.5 mol L^{-1} $NaHCO_3$; $Pi_{HID-0.1}$ and $Po_{HID-0.1}$: inorganic and organic P extracted with 0.1 mol L^{-1} NaOH; P_{HCl}: phosphorus extracted with 1.0 mol L^{-1} HCl; $Pi_{HID-0.5}$ and $Po_{HID-0.5}$: inorganic and organic P extracted with 0.5 mol L^{-1} NaOH; $P_{residual}$: phosphorus extracted with concentrated H_2SO_4+30% H_2O_2 and saturated magnesium chloride. Sequential fractionation in all sites was performed by a methodology proposed by Hedley, Stewart, & Chauhan (1982) and modified by Condron, Goh, & Newman (1985).

Supplementary References

Cherubin, M. R., Franco, A. L., Cerri, C. E., Karlen, D. L., Pavinato, P. S., Rodrigues, M., ... Cerri, C. C. (2016). Phosphorus pools responses to land-use change for sugarcane expansion in weathered Brazilian soils. *Geoderma*, **265**, 27-38.

Condron, L. M., Goh, K. M., & Newman, R.H. (1985). Nature and distribution of soil phosphorus as revealed by a sequential extraction method followed by ^{31}P nuclear magnetic resonance analysis. *European Journal of Soil Science,* **36(2)**, 199–207.

Hedley, M. J., Stewart, J. W. B., & Chauhan, B. (1982). Changes in inorganic and organic soil phosphorus fractions induced by cultivation practices and by laboratory incubations. *Soil Science Society of America Journal,* **46(5)**, 970-976.

Soltangheisi, A., Santos, V. R., Franco, H. C. J., Kolln, O., Vitti, A. C., Santos Dias, ... Pavinato, P. S. (2019). Phosphate sources and filter cake amendment affecting sugarcane yield and soil phosphorus fractions. *Revista Brasileira de Ciência do Solo.* **43:e0180227**.
